# The first complete chloroplast genome of *Coptis quinquesecta,* a critically endangered medicinal plant in China

**DOI:** 10.1080/23802359.2018.1450684

**Published:** 2018-03-14

**Authors:** Yunyan Zhang, Jiaxian Dong, Yu Feng, Zhongsheng Wang, Pan Li

**Affiliations:** a Key Laboratory of Conservation Biology for Endangered Wildlife of the Ministry of Education College of Life Sciences, Zhejiang University, Hangzhou, China;; b College of Life Sciences, Nanjing University, Nanjing, China

**Keywords:** Endangered plant, phylogenomics, ranunculaceae, ranunculoideae

## Abstract

*Coptis quinquesecta*, serving as a valuable medicinal plant, is a critically endangered and national key protected species of China. In the past, researches regarding this species mainly focused on its medicinal ingredients, whereas little effort has been made on its genetic information. Here we determined the first complete chloroplast genome of *C. quinquesecta* using genome skimming approach. The cp genome was 154,549 bp long, with a large single-copy region (LSC) of 84,409 bp and a small single-copy region (SSC) of 17,256 bp separated by a pair of inverted repeats (IRs) of 26,442 bp. It encodes 113 unique genes, including 79 protein-coding genes, 30 transfer RNA genes, and four ribosomal RNA genes. Besides, we reconstructed the phylogeny of Ranunculaceae based on the previously reported cp genomes of related taxa. A maximum likelihood (ML) phylogenetic analysis strongly supported the monophyly of Hydrastidoideae, Coptidoideae, and Thalictroideae, while Ranunculoideae was found to be paraphyletic.

*Coptis quinquesecta* W.T. Wang (Ranunculaceae) is a perennial herb scattered at altitudes of 1700–2500 m in Southeast Yunnan Province, China. It is characterized by the yellow rhizome and 5-sect basal leaves, which distinguishes it from other *Coptis* species. Meanwhile, treated as traditional Chinese medicine, it possesses the protoberberine alkaloids such as berberine and coptisine. These active compounds can be used for anti-viral, anti-inflammatory, and anti-microbial treatments due to their functions of dispelling dampness, removing toxicosis, and aiding detoxification (Yang et al. 2005; Zhang and Zhang 2006). However, owing to the human over-exploitation and habitat destruction, the population size of *C. quinquesecta* have dramatically decreased, causing the state of ‘critically endangered’ (CR) categorized by the Chinese Plant Red Book (Grade II Key protected Wild Plant; Xiong et al. 2011). By taking advantages of next-generation sequencing technologies that efficiently provide the chloroplast (cp) genomic resources of our interested species, we can rapidly access the abundant genetic information for conservation genetics (Hao et al. 2011). In this study, we reported and characterized the complete cp genome of *C. quinquesecta* based on the Illumina paired-end sequencing data. Furthermore, we reconstructed the phylogeny of Ranunculaceae employing the published related species’ cp genome sequences. These newly developed resources here will greatly contribute to the conservation of this endangered species.

Total genomic DNA was extracted from silica-dried leaves of *C. quinquesecta* collected from Jinping County, Yunnan Province, China (22°78′N, 103°23′E) using a modified CTAB method (Doyle and Doyle 1987). Voucher specimen of *C. quinquesecta* (LP174738) was deposited in the Herbarium of Zhejiang University (HZU). DNA libraries preparation and pair-end 125 bp read length sequencing were obtained on the Illumina HiSeq 2500 platform in Beijing Genomics Institute (Wuhan, China). About 3.8 Gb of raw data were firstly trimmed to remove low-quality bases (Phred score <30) by CLC-quality trim tool. Secondly, filtered reads were assembled into contigs using CLC Genomics Workbench 8 (Qiagen, Valencia, CA). Then, all the contigs were aligned to the reference cp genome of *Coptis chinensis* (KY120323; He et al. 2017) using BLAST (NCBI BLAST v2.2.31) search and the draft cp genome of *C. quinquesecta* was constructed by connecting overlapping terminal sequences in Geneious v10.0.5 software (Biomatters Ltd, Auckland, New Zealand). Finally, clean reads were re-mapped to the draft genome and yielded the cp genome sequence of *C. quinquesecta*. Gene annotation was performed via the online program Dual Organellar Genome Annotator (DOGMA; Wyman et al. 2004), and the cp genome physical map was drawn by Organellar Genome DRAW (OGDRAW; Lohse et al. 2007) with subsequent manual editing.

The complete cp genome of *C. quinquesecta* was 154,549 bp long and was deposited in GenBank with the accession number MG585353. Akin to other angiosperms, the cp genome of *C. quinquesecta* exhibited a typical quadripartite structure, consisting of a pair of inverted repeat regions (IRs with 26,442 bp) divided by two single-copy regions (LSC with 84,409 bp; SSC with 17,256 bp). The overall GC content of the total length, LSC, SSC, and IR regions was 38.3%, 36.4%, 32.2%, and 43.1%, respectively, which was similar to other taxa in Ranunculaceae (Park et al. 2017). Besides, there were a total of 113 unique genes, including 79 protein-coding genes, 30 tRNA genes and 4 rRNA genes, respectively. Among these genes, nine protein-coding genes and six tRNA genes contained a single intron, while three protein-coding genes possessed two introns. The gene *rps12* was trans-spliced; the exon at the 5′ end was located in the LSC region, however, the 3′ exon and intron were located in the IR regions. Moreover, the ^ψ^*ycf1* and ^ψ^*rps19* were identified as pseudogenes because of the partial duplication.

Furthermore, we reconstructed the phylogeny of Ranunculaceae based on the complete cp genome sequences of twelve Ranunculaceae species and two outgroup taxa, employing the GTR + G + I model and 1000 bootstrap replicates under the maximum-likelihood (ML) inference in RAxML-HPC v.8.2.10 on the CIPRES cluster (Miller et al. 2010). Our phylogenetic tree showed a better resolution of the subfamilies, with full support at all the nodes. Subfamilies Hydrastidoideae, Coptidoideae and Thalictroideae were monophyletic, while subfamily Ranunculoideae was paraphyletic, because tribe Adonideae was sister to Thalictroideae (Figure 1). This result was consistent with the most recent phylogenetic study on Ranunculaceae (Cossard et al. 2016). Overall, our data will largely enrich the genetic information of *C. quinquesecta* and facilitate future studies on conservation genetics.

**Figure 1.**
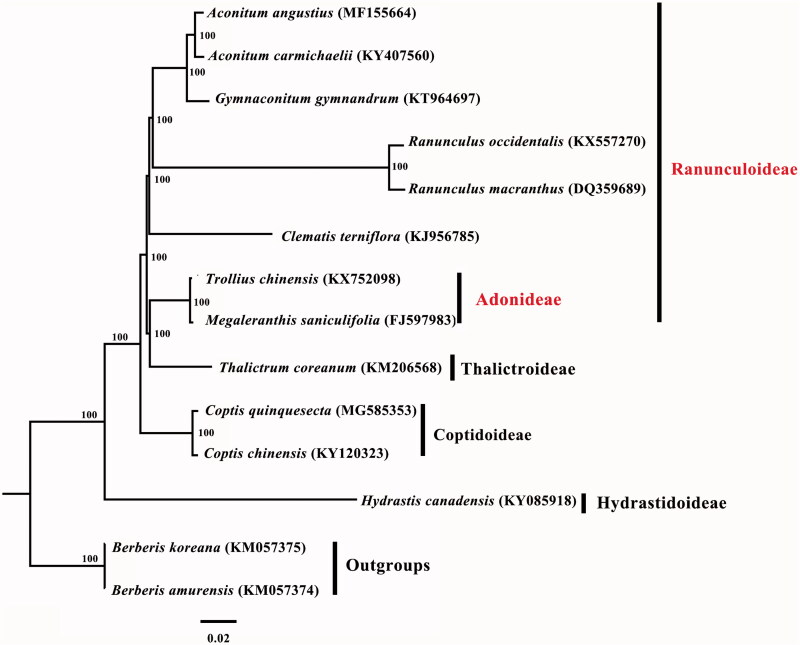
Phylogenetic tree reconstruction of twelve taxa of Ranunculaceae and two outgroups using ML method. Relative branch lengths are indicated. Numbers near the nodes represent ML bootstrap values.
